# Differences in phenology, daily timing of activity, and associations of temperature utilization with survival in three threatened butterflies

**DOI:** 10.1038/s41598-022-10676-0

**Published:** 2022-05-09

**Authors:** Markus Franzén, Yannick Francioli, John Askling, Oskar Kindvall, Victor Johansson, Anders Forsman

**Affiliations:** 1 Center for Ecology and Evolution in Microbial Model Systems, EEMiS, Department of Biology and Environmental Science, Linnaeus University, 391 82 Kalmar, Sweden; 2 Calluna AB, Linköpings slott, 582 28 Linköping, Sweden; 3 Department of Physics, Chemistry and Biology (IFM), Linköping University, 581 83 Linköping, Sweden

**Keywords:** Biodiversity, Climate-change ecology, Conservation biology, Evolutionary ecology, Grassland ecology

## Abstract

We used observational data collected during a mark-recapture study that generated a total of 7503 captures of 6108 unique individuals representing three endangered butterfly species to quantify inter-and intraindividual variation in temperature utilization and examine how activity patterns vary according to season, time of day, and ambient temperature. The Marsh Fritillary, the Apollo, and the Large Blue differed in utilized temperatures and phenology. Their daily activity patterns responded differently to temperature, in part depending on whether they were active in the beginning, middle or end of the season, in part reflecting interindividual variation and intraindividual flexibility, and in part owing to differences in ecology, morphology, and colouration. Activity temperatures varied over the season, and the Apollo and the Large Blue were primarily active at the highest available ambient temperatures (on the warmest days and during the warmest part of the day). The Marsh Fritillary was active early in the season and decreased activity during the highest temperatures. The relationship between individual lifespan and the average temperature was qualitatively different in the three species pointing to species-specific selection. Lifespan increased with an increasing range of utilized temperatures in all species, possibly reflecting that intra-individual flexibility comes with a general survival benefit.

## Introduction

**Figure 1:**
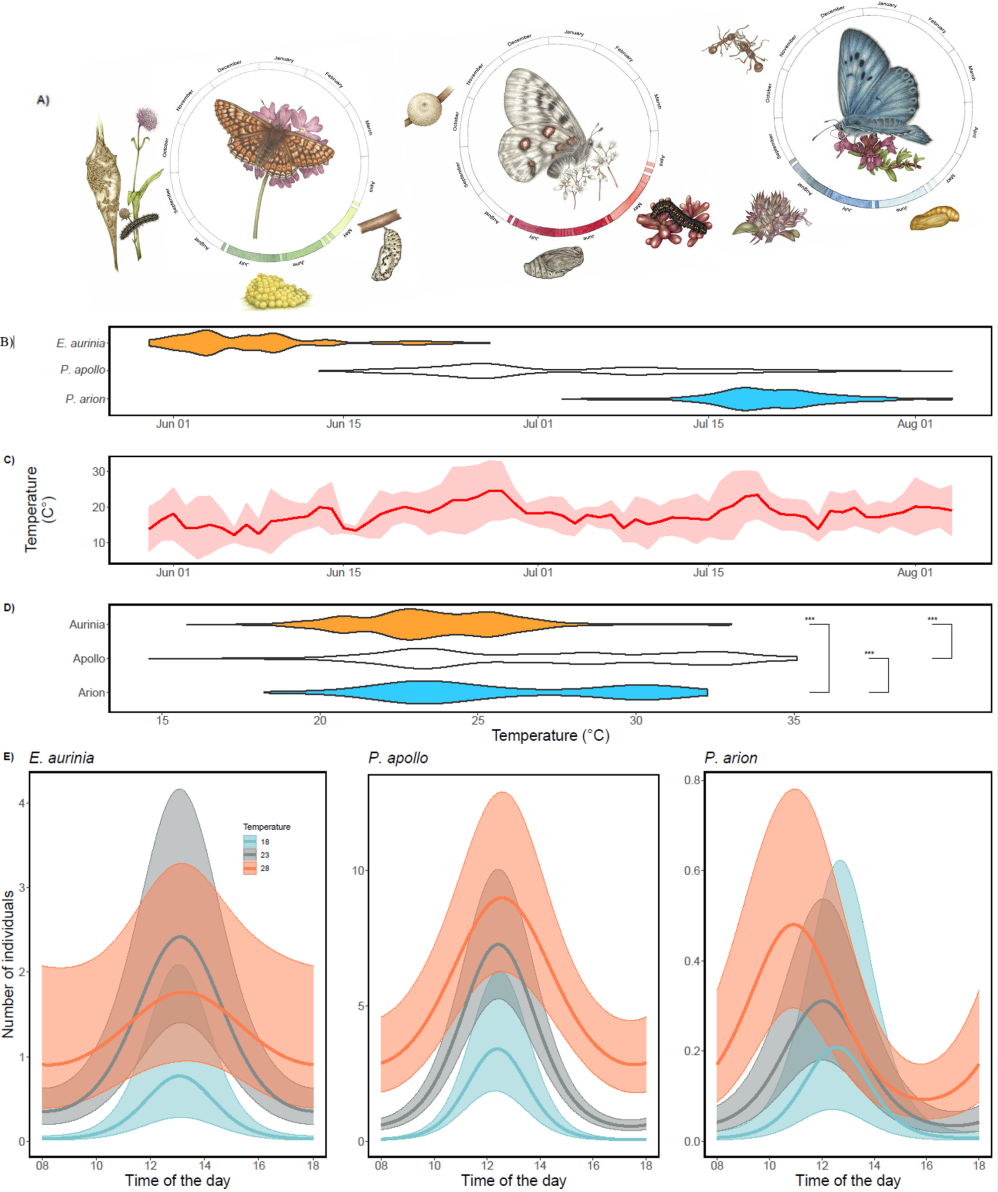
Seasonal and daily activity of three butterfly species in relation to temperature. (**A**) Life cycles of the study species Marsh Fritillary (*Euphydryas aurinia*), Apollo (*Parnassius apollo*)*,* and Large Blue (*Phengaris arion*) Drawings Emma Tinnert. (**B**) Phenology (violin plots) of each species based on the distribution of individuals caught across the flying period. (**C**) Variation in average daily ambient temperature (50 cm above ground, in the shade) over the sampling season, the standard deviation in pink. (**D**) Temperature niche of each species. (**E**) Comparable daily activity patterns at different ambient temperatures (18, 23, and 28 °C). The predicted number of butterfly individuals as estimated by generalized linear regression is shown (Table 1) (**B**).

Ongoing climate change imposes severe threats on biodiversity, ecosystem functioning, and human well-being worldwide^1–3^. This calls for an increased understanding of whether and how individuals, populations, and species cope with and are affected by changing and extreme temperatures. To enable the identification of underlying mechanisms, allow for reliable scenarios of future change, and assess the generality and reproducibility of research findings, it is also essential that the responses of species with different characteristics are simultaneously studied and systematically compared^4,5^. In response to increasing temperatures, species can move to colder areas^2,6,7^ by shifting distributions northwards (in the northern hemisphere) or to higher altitudes^4,8,9^. Besides large and small scale changes in species distributions, phenology changes are well-documented responses to elevated temperatures, with many species advancing their activity such that they become active earlier (or later) in the year^10–12^. Such phenology shifts may represent non-genetic responses in the form of developmental plasticity and phenotypic flexibility that has the potential to buffer against environmental change and uncertainty^4^. However, species and populations may also adapt to changing temperatures via genetically based evolutionary shifts in the shape of the reaction norm linking performance to temperature^1,13^. These can manifest as changes in the location of the optimal temperature or in the width of the thermal performance curve (see Fig. 1 in^14^).

Lepidopterans comprise a phylogenetically and functionally diverse, geographically widespread, and ecologically important group of animals that for long have attracted attention by ecologists and evolutionary biologists^15,16^. Being ectothermic, they are particularly well suited for studying responses to variation and change in temperature, both at different spatial and temporal scales and at different levels of organization, from behavioral and physiological responses of individuals to community-wide shifts in species composition and trait values^17^. For example, evidence is mounting that variation in range shifts, and phenology in response to climate change is associated with species traits^18,19^. However, species-level responses represent the joint outcome of the decisions and relative fitness of individuals. That temperature change may differentially affect different life cycle stages, from the egg-laying female to egg, larva, pupa, and imago, potentially complicating the situation further. For example, increased nitrogen loads may cause the shadowing of microclimatic conditions, and even in times of climate warming, the larvae of some butterfly species may suffer from lower temperatures on the ground^20^.

Adult insects, including butterflies, use a suite of physiological and behavioral adjustments to regulate body temperature, and their ability to maintain favorable temperatures is affected by several phenotypic traits, including body size and coloration^21–23^. Analogous to species range shifts, individuals can seek shade and select microhabitats with thermal properties that match their phenotypes and temperature preferences^24^. Analogous to phenology shifts, individuals can cope with unfavorable temperatures and avoid overheating by advancing or delaying the timing of their daily activities^25^. In principle, such intra-individual behavioral adjustments represent a form of phenotypic flexibility that can be targeted by natural selection and potentially undergo evolutionary shifts^26^. However, the evidence for this type of genetically based adaptation to rising and variable ambient temperatures is limited^25,27^. In particular, studies that quantify individual variation and estimate the magnitude and direction of selection on behavioral and physiological traits that influence the ability to cope with warm and changing thermal conditions are rare^28^. However, comparisons and knowledge of thermoregulatory mechanisms, seasonal and daily activity patterns, and how natural selection acts on thermal behaviors in different species can help assess their vulnerability to global warming^22^.

Here we studied how three globally endangered butterfly species (the Marsh Fritillary (*Euphydryas aurinia*), the Apollo *(Parnassius apollo*), and the Large Blue (*Phengaris arion*)) that differ in body size, color pattern, life-history, thermal niches, and phenology adjust their daily timing of activity depending on season and in response to changes in ambient temperature. We selected these three different and distantly related species in part because they are threatened and in part, because they differ in morphology, physiology, life-style and microhabitat use. Yet, they co-occur within our study site on northern Gotland (see below) thus allowing for powerful comparisons of their responses to temperature and temperature change. In addition to the between-species comparisons, we quantify both inter-and intraindividual variation in temperature utilization and explore how these are associated with the lifespan of individuals. Specifically, we examine whether and how: (i) the thermal niche changes over the season within each species, (ii) phenology differs between the three species, (iii) the thermal niche varies among the three species according to their phenology, (iv) the daily timing of activity is associated with ambient temperature, (v) the shape of the behavioral reaction norm linking activity to temperature is different for individuals that emerge and are active in the beginning, middle, or end of the flight period, and (vi) how thermal niches vary within species (are there differences between sexes, and do some individuals within each sex consistently utilize relatively low temperatures and others consistently utilize relatively high temperatures?). Finally, (vii) to evaluate natural selection and the potential for adaptive responses, we examine whether there is an association between the variation in the thermal preferences (utilized temperatures) and the lifespan of individuals. For this, we used a unique dataset of 7719 individually marked butterflies together with high-resolution temperature logger data.

## Materials and methods

### Description of studied species

The Marsh Fritillary, the Apollo butterfly, and the Large Blue are univoltine, rapidly declining species included in EU's Habitats Directive (Council Directive 92/43/EEC), protected by law within the EU and red-listed in Europe^16^.

The large blue is a small shiny blue butterfly with dark spots on the forewing measuring from 32 to 42 mm. It has a western Palaearctic distribution across large parts of Europe to China. It is a very local and thermophilic species associated with dry grasslands. In the study area the species occurs in dry unfertilized calcareous grasslands and alvar that in most places remain naturally open due to the poor soil and slow accumulation of humus. The butterfly visits many different flowers for nectar, while the larva specialises in feeding only *Thymus serpyllum* (in our study area). Females lay their eggs on the host plant. The butterfly is univoltine and active from July to August^29^. The larva hatches in August and is adopted by *Myrmica* ants to their nests, and the butterfly larvae continue feeding on ant broods as a parasite^30^. The larva hibernates in the ant nests and pupates in June. Parasitic wasps can attack the larvae, and the adult butterflies are sometimes caught by dragonflies and spiders^31^.

The Apollo is a large white butterfly from 73 to 87 mm with black and red elements of variable size on the wings. It has a scattered distribution across large parts of Europe to China. This iconic butterfly primarily inhabits areas where bare rocks and vegetation free surfaces occur. In the study area, the species occurs on open alvar terrain that in most places remains naturally open due to the lack of vegetation establishment on the limestone. Since the 1950ies, this threatened butterfly has disappeared from large areas and the remaining populations are very isolated. The butterfly is a keen visitor of many different flowering plant species for nectar, while the larva only feeds on *Sedum album* (in our study area). Females lay their eggs on shrubs, bushes and different vascular plants. The butterfly is univoltine and active from June to August^29^. The eggs overwinter, and the dark and orange conspicuously looking larvae hatch March/April and pupate in June.

The marsh fritillary is a butterfly with an average size of 33 to 48 mm in wingspan and orange to brown, with black dots. It is distributed in northern Africa and across large parts of Europe to China. It occurs very locally and is inhabits fens and ungrazed grasslands in the study area^32,33^. The butterfly is visiting different plant species for nectar, and the larva is specialized to only feed on *Succisa pratensis* (in our study area). Females lay egg batches under the leaves of the host plant. The butterfly is univoltine and active from May to June, and the larva hatch in July/August^29^. The larvae live gregariously and spin a silk web, by binding together leaves of the foodplant, in which they live and feed. After changing into the 4th instar the larva hibernates low down in vegetation and start to feed again in spring. The fully-grown larvae pupate in May/June.

### Description of the study-area

The study area of 60 km^2^ (10 km × 6 km) is located close to Slite on the island of Gotland in the Baltic Sea (Fig. S1), Sweden (midpoint of the area: 57°69′N 18°69′E), where the butterflies occur across large areas. This is one of the last remaining areas in Europe that support viable populations of these butterflies within the same landscape. The climate is typical with cool summers and cold and rainy winters, and the average temperature is 7.2 °C (max in July with an average daily of 16.6 °C, and coldest in Feb with − 2.1 °C), and average yearly rainfall is 524 mm, and July to January has more rain per month (> 50 mm compared to February to June (< 33 mm). The study area is very diverse and captures 15 habitat types of the Habitats Directive. Some parts of the landscape have been extensively grazed by livestock, a farming practice that has intensified since 2000. Natural old forests, dominated by pine woodlands ranging from very open woodlands with single old trees on thin soils to dense forests on more rich soils, are scattered throughout the area. Agricultural fields with the use of pesticides, herbicides and inorganic fertilizer are delimiting the study area towards West and North East.

### Data collection

We individually captured and marked all three butterfly species that we could catch from the 30th of May to the 4th of August 2020 (Fig. S2). This period covered all three studied species' start and end flight periods. Fieldwork was performed between 8 a.m. and 6 p.m. with suitable weather by up to ten persons. For each captured butterfly, species, sex, position, and time were registered. Butterflies were registered along irregular routes focused on covering all suitable habitats in focal areas within the study landscape (Fig. S3). Their lifespan was calculated as the time interval between the first and last capture. Surveys were not performed in unfavorable weather conditions such as rain (within one h after rainfall) and temperatures below 14 °C. The temperature was recorded at intervals of 1 h (even intervals) using the HOBO MX2202 pendant wireless temperature/light data loggers placed in shade 0.5 m above ground (Fig. S4). The temperature logger was placed in the core of the study area closest to the populations of the three butterfly species (Latitude: 57.72092, Longitude: 18.6831). We had access to ten other temperature logging stations distributed across our study area, revealing ambient air temperatures to be highly correlated (Table S1). Individual captures for each species were linked to the temperature data by 1-h bins (from 8:00 to 18:00).

### Statistical analysis

For all statistical analyses, the R software was used^34^. To examine whether there is variation in utilized temperature among individuals within each species, we built General Linear Mixed Models (GLMMs). We tested two models with the realized temperature and sex as the response variable, the first one with butterfly individuals as a random term, against the null model, comparing the log-likelihood. We included sex as a factor in the models to test if individuals within each sex consistently utilize relatively lower temperatures and others utilize relatively higher temperatures.

To examine whether and how the daily timing is associated with ambient temperature while accounting for the cyclic nature of the time of the day, we used a trigonometric regression approach^35^. The time of the day was converted to radian (8:00 being 0 and 18:00 being 2*pi), allowing it to fit as a sine and cosine wave^36^ in a linear regression model. Similarly, to account for the seasonal activity changes, we converted the day of capture to radian, with 0 as the first day the species was recorded and 2*pi as the last day). The other continuous variable, the temperature, was scaled for all regression analyses. To control for possible effects of sex, we included sex as a fixed factor in the model. We also controlled the varying sampling effort by adding the number of persons involved in sampling as a fixed effect in all regression models.

To analyze how butterfly activity changes over the day, we first summed the number of individuals captured for each species in 1-h bins from 8:00 to 18:00 and then used a generalized linear model (GLM) with the number of individuals caught as the response variable and a negative binomial error structure. As explanatory variables (fixed effects), we included sex of the individual (to evaluate and account for differences between males and females), the temperature (with a linear term and a quadratic term of degree two), the time of the day (as sine and cosine wave) and its interaction with the linear temperature term, the day of the season (as sine and cosine wave) and the number of persons involved in the sampling.

To examine whether and how the thermal niche changes over the season within each species, we analyzed the temperature utilization relative to the time of the flying season with GLMs. The response variable was the presence/absence of the species in each daily hour bin. The explanatory variables were the temperature (with a linear term and the squared term to evaluate both linear and curvilinear associations), the day of the season (as sine and cosine wave), and their interaction and sex as a fixed factor. We did not evaluate the effects of interactions between sex and the other explanatory variables to avoid over-parametrization of statistical models and because sample sizes were very different for males and females, both of which may generate unreliable results and conclusions. However, to assess whether results were qualitatively similar or different in the two sexes, we also performed separate analyses for males and females. To test whether or not species were consistently flying above the average available temperature, we first computed the daily difference between the average available and average used temperature and then tested if the mean distribution of these differences is significantly different from zero using a one-sample *t*-test.

**Figure 2:**
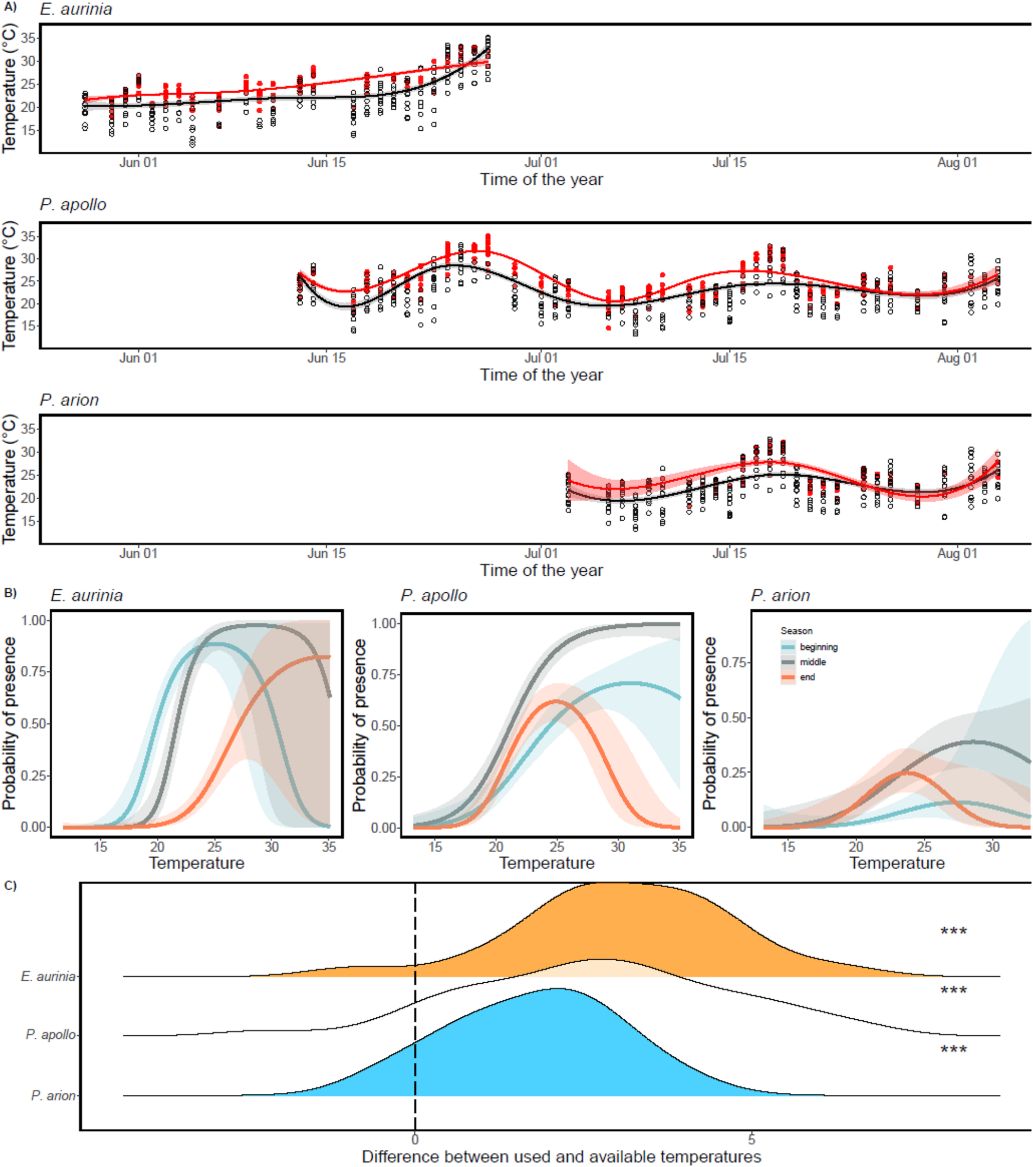
Seasonal variation in realized thermal niches to available ambient temperatures for three butterfly species. (**A**) Ambient temperatures when butterflies were active/caught (shown in red) and temperature recordings when no butterflies were found (open black circles). The fitted spline curves denote occupied temperatures (red) and all available temperatures (black). (**B**) Comparisons of reaction norms linking activity (probability of presence) to available ambient temperature in the beginning, middle, and end of the activity period for each species (see methods). The interaction is statistically significant for Marsh Fritillary (*Euphydryas. aurinia)* and Apollo (*Parnassius apollo*). (**C**) Distribution of the averaged daily difference between used and available temperatures for each species. Significance levels from one-sample *t*-test. *** denotes *p* < 0.0001.

We used ggplot^37^ for spline curve-fitting technique to display available and realized temperatures along the flying season of each species, with 4, 7, and 4 knots for the Marsh Fritillary, the Apollo, and the Large Blue, respectively. For illustrative purposes (Fig. 2B), the season’s beginning, middle, and end were set as 2020-06-03, 2020-06-11, and 2020-06-20 for Marsh Fritillary; 2020-06-24, 2020-07-09, and 2020-07-23 for Apollo; and 2020-07-10, 2020-07-19, and 2020-07-29 for the Large Blue butterfly.

To examine the association between the individual variation in temperature utilization and lifespan, we used GLMs. We treated lifespan as the response variable. We included sex, median realized temperature (with a linear term and a quadratic term), realized temperature range, and date of the first capture as explanatory variables.

The package glmmTMB was used for the GL(M)Ms that assumed a negative binomial error with a log link function structure for the activity pattern, binomial for the temperature niche models, and a Gaussian error structure for the lifespan and individual thermal niche models. Model marginal effects and confidence intervals (CIs) were calculated using the ggeffects package^38^. Raw data plots illustrating the distribution of the analyzed data are presented in Fig. S5–S7.

## Results

In 2020, we captured a total of 6108 (1430 Marsh Fritillary, 4518 Apollo, and 160 Large Blue) unique butterfly individuals. The recapture rate was 0.34, 0.10, and 0.28 for a total of 7503 captures (2164, 5109, and 230, respectively). The female frequency was 0.25, 0.29 and 0.20, respectively. Individuals of Marsh Fritillary, Apollo, and Large Blue butterflies were recaptured up to a maximum of 4, 5, and 4 times, respectively. The number of individuals recaptured at least three times was 124, 59, and 8, and at least four times was 35, 14, and 1, respectively.

### Phenology

There were pronounced differences in phenology between the species. The Marsh Fritillary was active first (the first day is 28th of May, the median day is 7th of June, and the last day is 27th of June), followed by Apollo, which had the most extended activity period (the first day is 13th of June, the median day is 29th of June, and last day is 4th of August), and the Large Blue (the first day is 3rd of July, the median day is 19th of July, and last day is 4th of August) (Fig. 1a, Fig. S8). The Marsh Fritillary and Apollo showed seasonal peaks at the beginning of their flying periods. In contrast, the Large Blue showed a peak in the middle of the flying period (Fig. 1a, Table 1).

**Table 1:** Predictors of the number of individuals, models output for each species.

Predictors	*E. aurinia*			*P. apollo*			*P. arion*		
	Est	SE	*p* value	Est	SE	*p* value	Est	SE	*p* value
Intercept	− 0.445	0.287	0.121	− 1.853	0.191	**< 0.001**	− 4.006	0.385	**< 0.001**
sin(Time of the day)	− 0.056	0.099	0.57	0.445	0.089	**< 0.001**	0.627	0.15	**< 0.001**
cos(Time of the day)	− 0.937	0.148	**< 0.001**	− 1.182	0.106	**< 0.001**	− 0.881	0.153	**< 0.001**
Temperature	0.842	0.127	**< 0.001**	1	0.099	**< 0.001**	0.702	0.186	**< 0.001**
Temperature^2^	− 0.527	0.091	**< 0.001**	− 0.198	0.068	**0.004**	− 0.089	0.131	0.494
Number of persons	0.152	0.046	**< 0.001**	0.394	0.03	**< 0.001**	0.203	0.058	**< 0.001**
Sex (female as reference	0.984	0.163	**< 0.001**	0.771	0.12	**< 0.001**	1.667	0.204	**< 0.001**
sin(Time of the year)	1.482	0.139	**< 0.001**	0.685	0.091	**< 0.001**	− 0.877	0.17	**< 0.001**
cos(Time of the year)	0.051	0.19	0.787	− 0.662	0.099	**< 0.001**	− 0.844	0.189	**< 0.001**
sin(Time of the day)*Temperature	0.008	0.113	0.945	− 0.253	0.085	**0.003**	0.146	0.145	0.316
cos(Time of the day)*Temperature	0.533	0.179	**0.003**	0.55	0.12	**< 0.001**	0.586	0.192	**0.002**

Estimate, CI, and *p* values from a GLM with a negative binomial error structure.

Significant values are in bold.

### Comparisons of temperatures during activity among species and individuals

The average ambient daily air temperatures fluctuated considerably over the season, ranging from 10.7 to 23.7 °C, and the minimum and maximum temperature recorded during the study was 11.8 °C and 35.1 °C, respectively (Fig. 1b). The butterflies were active only during a restricted range of the available temperatures. The differences in utilized temperatures between species corresponded overall with the differences in phenology, being lowest (mean ± S.D.) 23.6 ± 2.5 °C for the Marsh Fritillary that appeared first in the season, 27.1 ± 4.3 °C for the Apollo and 25.7 ± 3.6 °C for the Large Blue (Fig. 1c). Although the utilized temperatures largely overlapped, particularly between the Apollo and the Large Blue, and were relatively broad, spanning ca 10–12 °C for each species, all pairwise differences between species were statistically significant (two-sample *t*-test) (Fig. 1c).

The broad range of utilized temperatures observed within each species was partly due to a combination of pronounced interindividual variation and high intra-individual flexibility. Data for individuals captured on three or more different days showed that there was also significant variation among individuals that belonged to the same sex, within all three species (Fig. S9). Part of the variation among individuals reflected differences between males and females, at least in the Marsh Fritillary, where females were active in higher temperatures than males (Fig. S10). Within each species, some individuals consistently utilized relatively low temperatures, whereas others consistently utilized intermediate or relatively high temperatures (effect of individual identity evaluated using log-likelihood ratio test, Marsh Fritillary: χ^2^ = 53.42, d.f. = 1, *p* < 0.0001; Apollo: χ^2^ = 23.18, d.f. = 1, *p* < 0.0001; Large Blue: χ^2^ = 2.98, d.f. = 1, *p* = 0.1126). The intraindividual temperature *range* during activity spanned on average ca 5 °C in all three species, but with considerable variability among individuals (Fig. S9). The intraindividual *median* temperature during activity also varied considerably among individuals, from 18 to 32 °C (*n* = 124) in the Marsh Fritillary, from 22 to 32 °C (*n* = 60) in the Apollo, and 23 to 29 °C (*n* = 8) in the Large Blue (Fig. S9).

### Comparisons of daily activity patterns in relation to ambient temperatures

All three species exhibited distinct daily activity patterns modified by ambient temperatures (Table 1, Fig. 1c). The activity of the Marsh Fritillary generally peaked around noon and was highest on days with intermediate temperature; on hot days (primarily towards the end of their activity period around midsummer, see Fig. 2a), they became less active in the middle of the day (avoiding the highest temperatures) and were instead more active in the morning and late afternoon, compared with colder days. The activity of the Apollo also peaked around noon, and its activity coincided with the highest ambient temperatures. On colder days, the Apollo showed a clear peak of activity in the middle of the day and very low activity in both the morning and the evening, while on very warm days, this species became active earlier in the morning and remained active later in the afternoon, compared with intermediate and cold days. The daily activity of the Large Blue reached a peak around noon on cold and intermediate days, was highest on warm days, and differed from the other two species in the sense that on warmer days, the activity peak of the Large Blue shifted and occurred earlier in the day (Fig. 1c).

### Utilized *versus* available temperatures

The comparisons of utilized and available temperatures show that both the seasonal phenology and the daily activity patterns of the three butterfly species were modified by environmental temperatures (Tables 1, 2, Figs. 1, 2). Although the temperatures at which butterflies were active consistently changed (increased, the Marsh Fritillary) or fluctuated (the Apollo and the Large Blue) over the season in concert with the variation in ambient temperatures, all three species were primarily active at the highest available ambient temperatures (on the warmest days and during the warmest part of the day), except towards the end of their respective activity periods and when the ambient temperatures were very high (Fig. 2).

**Table 2:** Predictors of species presence, models output for each species.

Predictors	*E. aurinia*			*P. apollo*			*P. arion*		
	Est	SE	*p* value	Est	SE	*p* value	Est	SE	*p* value
Intercept	− 1.235	0.389	**0.002**	− 0.75	0.115	**< 0.001**	− 3.46	0.316	**< 0.001**
Temperature	50.975	8.471	**< 0.001**	30.993	3.184	**< 0.001**	17.707	6.32	**0.005**
Temperature^2^	− 28.998	13.167	**0.028**	− 20.806	3.977	**< 0.001**	− 25.506	9.977	**0.011**
sin(day of the season)	1.712	0.32	**< 0.001**	0.087	0.117	0.459	− 0.476	0.289	0.1
cos(day of the season)	− 0.862	0.346	**0.013**	− 1.313	0.158	**< 0.001**	− 0.955	0.298	**0.001**
Sex (Female as reference)	0.51	0.217	**0.019**	0.264	0.14	0.06	1.78	0.264	**< 0.001**
Temperature * sin(day of the season)	− 17.823	9.475	0.06	4.145	4.43	0.35	10.552	8.311	0.204
Temperature * sin(day of the season)	− 12.666	9.963	0.204	14.234	4.548	**0.002**	11.343	11.444	0.322
Temperature * cos(day of the season)	− 29.786	8.654	**< 0.001**	− 18.924	6.064	**0.002**	− 7.211	8.195	0.379
Temperature^2^ * cos(day of the season)	10.518	13.325	0.43	− 17.666	7.416	**0.017**	− 12.95	11.243	0.249

Estimate, CI, and *p* values from a GLM with a binomial error structure.

Significant values are in bold.

### Species-specific associations of utilized temperatures with lifespan

**Figure 3:**
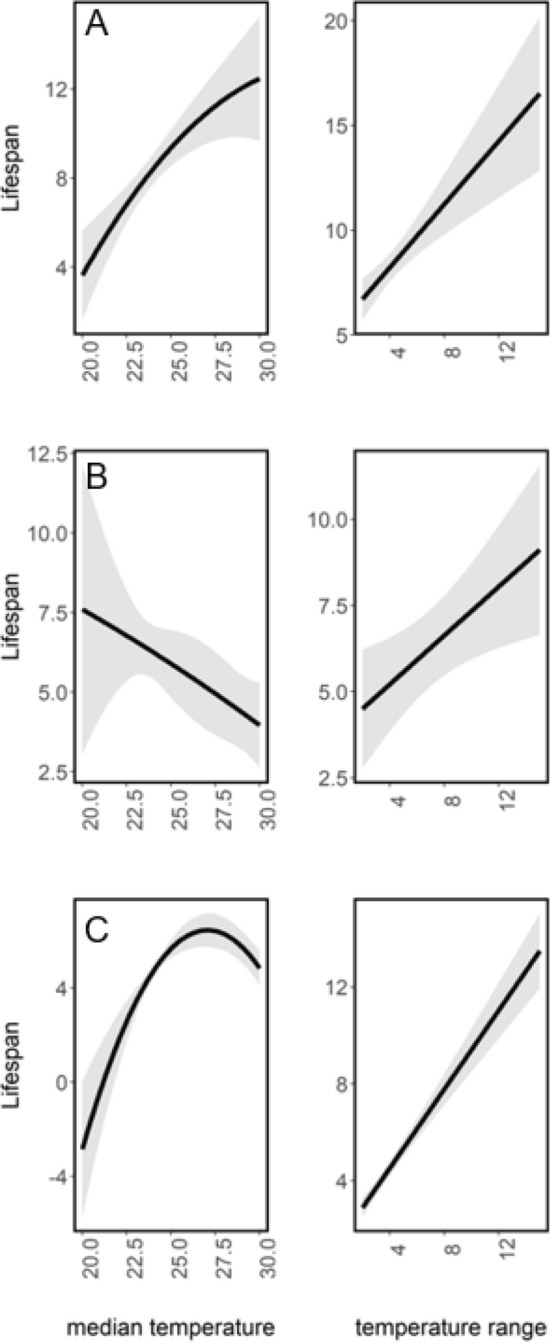
Associations of variation in thermal utilization with individual lifespan in three butterfly species. Thermal utilization is inferred from the median (left panels) and the range (max–min, right panels) of ambient temperatures, respectively, at the time of collection. Lifespan is inferred from retention time in the population. Data for (**A**) Marsh Fritillary (*Euphydryas aurinia*), (**B**) Apollo (*Parnassius apollo*)*,* and (**C**) Large Blue (*Phengaris arion*) butterfly individuals captured on at least three separate days. Effects plots based on results from the general linear models are shown (see Table 3).

The relationship between the lifespan and the temperatures utilized by individuals was qualitatively different in the three species (Table 3). In the Marsh Fritillary, lifespan increased with increasing thermal preferences and increased range of utilized temperatures after statistically factoring out the effect of the date of the first capture (Table 3, Fig. 3a). In the Apollo, lifespan decreased with increasing thermal preferences and increased with an increasing range of utilized temperatures (Table 3, Fig. 3). There was a curvilinear (quadratic) association between lifespan and thermal preferences in the Large Blue, indicative of stabilizing selection, and a positive linear effect on the lifespan of the range of utilized temperatures (Table 3, Fig. 3). The correlation between the range of utilized temperatures and the number of capture events was weak at best (*Marsh Fritillary*: *r* = 0.18, *p* = 0.041, *n* = 124; *Apollo*: *r* = 0.16, *p* = 0.23, *n* = 59; and *Large Blue*: *r* = 0.06, *p* = 0.89, *n* = 8) (Table S2), suggesting that the increase in lifespan with increasing temperature range observed in all three species is not a spurious outcome but reflective of a true underlying association.

**Table 3:** Associations of individual variation in lifespan with thermal utilization and date of the first capture in three butterfly species. Lifespan is inferred from retention time in the population. Thermal utilization is inferred from the median and the range (max–min) of ambient temperatures, respectively, at the time of collection. Data for (a) Marsh Fritillary (*Euphydryas aurinia*), (b) Apollo (*Parnassius apollo*, and (c) Large Blue (*Phengaris arion* butterfly individuals captured on at least three separate days. Both linear (temperature) and nonlinear (quadratic, temperature^2^) associations with median temperature were evaluated.

Predictors	*E. aurinia*			*P. apollo*			*P. arion*		
	Est	SE	*p* value	Est	SE	*p* value	Est	SE	*p* value
Intercept	60.473	11.99	**< 0.001**	9.69	6.094	0.112	− 194.815	9.645	**< 0.001**
Median temperature	20.817	4.811	**< 0.001**	− 8.134	2.738	**0.003**	1.842	0.238	**< 0.001**
Median temperature^2^	− 5.789	3.467	0.095	− 0.229	3.049	0.94	− 2.188	0.192	**< 0.001**
Temperature range	0.755	0.169	**< 0.001**	0.355	0.135	**0.009**	0.816	0.031	**< 0.001**
Date of the first capture	− 0.346	0.075	**< 0.001**	− 0.036	0.031	0.256	1.004	0.047	**< 0.001**
Sex (female as reference)	− 1.568	0.918	0.088	0.96	1.057	0.363	− 7.544	0.189	**< 0.001**

Estimate, CI, and p values from a GLM with Gaussian error structure.

Significant values are in bold.

## Discussion

Mapping of thermal environments together with high-resolution data on activity patterns and knowledge of how natural selection acts on thermal behaviors in different species can inform about variation in sensitivity to temperature change and ultimately help assess their vulnerability to global warming. This study aimed to investigate and compare the patterns, causes, and consequences of variation in phenology, daily timing of activity, and temperature utilization in three endangered butterfly species. We demonstrate that the Marsh Fritillary, Apollo, and the Large Blue differed in utilized temperatures and phenology and that their daily activity patterns responded differently to temperature. We argue that these differences between the species in part depend on whether they were active in the beginning, middle, or end of the season, and in part, reflect a combination of interindividual variation and intraindividual flexibility. In addition, differences between the three species in ecology, body size (wingspan), and coloration may have contributed to the observed dissimilarities, as discussed further below. We also show that activity temperatures varied over the season, with two species (the Apollo and the Large Blue) being primarily active on the warmest days and during the warmest part of the day, except on very hot days and towards the end of their respective activity periods. The Marsh Fritillary that was active early in the season instead decreased activity during the highest temperatures. Our study design allowed for novel insights into the fitness consequences of temperature utilization in butterflies. The demonstration that some individuals were consistently active at relatively low and others at relatively high ambient temperatures points to pronounced interindividual variation (within each of the two sexes) in thermal biology in all three species. The associations with individual lifespan illustrate this variability’s ecological importance, and the relationship between lifespan and average utilized temperature was qualitatively different in the three species (positive, curvilinear, and negative). We argue that this provides rare evidence of species-specific selection and fitness consequences of thermal preferences in butterflies. By contrast, the finding that lifespan invariably increased with an increasing *range* of utilized temperatures in all three species indicates that high intra-individual flexibility (i.e., broad activity temperatures) comes with a general survival benefit. These findings and their implications are discussed in greater detail below.

### Do the differences in temperature utilization represent consequences of different phenotypes or species-specific adaptations to phenology?

Ectothermic animals such as insects may vary in temperature preferences as well as in their ability to regulate body temperature using a plethora of structural, morphological, physiological, and behavioral traits, including the selection of microhabitats with thermally suitable conditions^22–24,39,40^. In keeping with this notion, we propose that the differences between species in temperature utilization, phenology, and viability selection indicated by our results may be partly attributable to the thermal significance of body size and coloration^22,41^. Previous studies report spatial and temporal community-wide shifts towards smaller and paler species of butterflies in warmer climates^27,42,43^. Our findings that the largest species in this study, the Apollo butterfly, was active during the warmest part of the season and generally utilized the highest ambient temperatures do not conform with this pattern. However, the Apollo is also light in color (Fig. 1), which reduces the risk of overheating^23,40^. This species also showed reduced activity at midday during the hottest periods and a reduction in lifespan with increased average temperature utilization, indicating that avoiding overheating was indeed important. This interpretation fits well with previous demonstrations of intrapopulation trait variation, pointing to correlated evolution of body coloration, behavior, and thermal physiology^40^. By comparison, the Marsh Fritillary and the Large Blue were active earlier and later in the season when it might be important to increase body temperature by sun basking, and they are both darker in color (Fig. 1).

Being holometabolous insects with complex life cycles, there is potential for selection at the larval stage to influence the evolution of phenology and temperature utilization. Both the Marsh Fritillary and the Apollo have black larvae (Fig. 1) active in early spring when ambient temperatures are low, and the ability to increase body temperature by basking might allow for faster growth and development^44,45^. The larvae of the Large Blue occupy subterranean ants’ nests where their yellowish color likely plays little or no role in temperature regulation^46^. However, firm conclusions regarding the driver of associations of larval coloration with temperature and phenology are further complicated because the evolution of larval coloration may also be influenced by selection to avoid detection or attack by predators^47,48^.

Insofar as the extensive inter- and intraindividual variation in temperature utilization observed within each species is genetic in origin (heritable), the differential viability selection on temperature utilization indicated by our results may contribute to the evolution and continued maintenance of species-specific thermal biology. There is little doubt that the variation in temperature utilization among individuals *within* species and sexes demonstrated by our results is partly attributable to phenotypic differences in body size and coloration that may influence the capacity for temperature regulation and partly attributable to developmental plasticity and phenotypic flexibility. For example. temperature utilization may differ between individuals that hatch early versus late in the season due to the temperature conditions experienced earlier in life^49^. Studies of other species of butterflies show that the temperature, humidity, and light conditions experienced during the larval or pupal stage can induce the development of different color patterns of the imagoes^50,51^. It is also conceivable that acclimatization and developmental plasticity may result in associations of developmental temperature with thermal preferences and the shape of reaction norms linking performance to body temperature in adults^52,53^. However, to our knowledge, it has not been investigated whether this applies to any of the butterfly species studied here.

### Why does phenology differ between the three species?

The substantial differences in phenology between the three species seemingly coincide with the variation in utilized temperatures. Notably, the Marsh Fritillary was active earliest in the season and utilized the lowest temperatures on average, compared with the other two species. Advanced phenology may be required to avoid overheating if the species are adapted to lower temperatures (low thermal optima). Conversely, their early adult flight period might have induced correlated microevolutionary shifts in behavior and thermal physiology such that they can better cope with lower temperatures^54^.

The differences may also reflect phenological synchrony between the butterflies and their respective host plants^55^. The butterfly species depend on their host plant for larvae development and nectar plants for adults. These plants are only available during certain time windows. The Marsh Fritillary larvae feed on *Succisa pratensis* from July to May that flowers in August/September^56^ and imagoes are nectaring from various flowers during the activity period. Apollo larvae feed on *Sedum album* from March to May, and the Large Blue larvae feed on *Thymus serpyllum* in August and forage on ant brood from September to May. Both the Apollo and the Large Blue feed almost exclusively on nectar from their host plants (e.g., *Thymus serpyllum* for the Large Blue, and *Sedum album* for Apollo) that flowers during the adult activity period. Besides searching nectar on their larval host plants, Apollo frequently visits the nectar-rich plants’ *T. serpyllum* and *Centaurea scabiosa* that have a longer flowering season and are available later in the season compared to the larval host plant *Sedum album *^29^. The phenological separation between butterfly species may lessen interspecific competition for nectar resources. In our study area, these three sympatric species dominate the butterfly community, and if their activity periods were completely overlapping, over-exploitation might impair their foraging performance^57^. One way to evaluate this competitive displacement hypothesis would be to compare the patterns reported here with the phenology and temperature utilization of the same species in areas where they are allopatric/do not coexist.

Whatever ultimate driver(s) contributed to their evolutionary origin, we suggest that the phenology differences between the three species are maintained by ongoing selection. In support of this interpretation, our analyses revealed statistically significant viability selection on the seasonal timing of activity in two of the studied species (Table 3). In the Marsh Fritillary, individuals with a relatively early flight period survived longer than their later conspecifics. In the Large Blue, individuals that were active relatively late in the season instead had a longer lifespan. Together with the documented selection on temperature utilization, this may contribute to phenological adaptations and continued evolutionary divergence of phenologies in response to warmer future temperatures.

### How will these species respond to continued climate change?

Theory and empirical evidence concur that the combination of large interindividual variation and high intra-individual flexibility observed in all three species may buffer the populations against environmental change and uncertainties, stabilize population dynamics, reduce the risk of local extinctions, facilitate the establishment of new populations, and promote evolvability^18,58–60^. Regarding the potential for evolutionary adaptations to increasing temperatures in the butterflies studied here, our results suggest that there exists variation in temperature utilization among individuals for selection to act upon in the populations of all three species. We cannot estimate the heritability of phenology or thermal traits from the data in this study. However, previous studies have revealed significant heritability estimates for thermal traits^61,62^. We observed associations of temperature utilization with lifespan indicative of selection in all three species. Given that all the requirements are met, we conclude that there is potential for adaptive responses and different microevolutionary shifts of thermal traits in the system studied here. Moreover, the general survival benefit of high intra-individual flexibility of activity temperatures will likely shield the populations against the erosion of genetic variation typically associated with directional selection^53^, thereby enabling future adaptive responses to continued climate change. The aforementioned directional viability selection on the seasonal timing of activity may also induce adaptive shifts in phenology. However, whether these responses will be sufficiently rapid to keep up with climate change is uncertain.

Regarding implications for population trends^22^, our results show that there were periods during the season and parts of days when stressful temperatures (too high or too low) suppressed activity, thereby likely constraining foraging and reproductive opportunities. However, firm projections require that the consequences of temperature change on other life-stages and other species with which the butterflies interact are also considered^63^. Maintaining phenological synchrony with host plants may be particularly important^55^.

In our study area, Marsh Fritillary decreased in occupancy by over 30% between 2018 and 2019 due to an extreme drought^32^. In Finland, a tenfold decline following the 2018 drought was observed in the closely related Glanville fritillary^64^. Previous studies have shown that *Large Blue* fluctuates dramatically in abundance^65^ and that populations often decrease following years with high temperatures and droughts^46^. Less is known about how the population dynamics of Apollo is affected by temperature, as it is very rare throughout the distribution^66^.

Regarding distribution shifts, the estimates of temperature utilization in our study are higher overall. Still, the differences *between* the three species are in qualitative agreement with the differences in thermal tolerances extracted from the species distribution areas^67^. Bladon, et al.^22^ report that butterfly species relying on microclimate selection have suffered larger spatial population declines over the last 40 years than those altering their temperature behaviourally. However, butterflies have generally declined in numbers and distribution throughout Europe^16^.

The consequences of changing temperatures go beyond the responses and species studied here. Butterflies interact with several other types of organisms, as competitors, pollinators, prey for spiders and birds, and even as predators (e.g., the Large Blue feeds on ants)^27^. There are thus many ways by which modifications in phenology, daily activity patterns, abundance, and distribution of these butterflies may cascade up and down in the ecosystems and affect the services they provide.

## Conclusion and future directions

In sum, our results demonstrate striking differences among the three butterfly species in phenology, utilized temperatures, how ambient temperatures modified daily activity patterns, and how the individual variation in utilized temperatures was associated with lifespan. This emphasizes the need to simultaneously study and compare the responses to temperature change of species with different characteristics. The responses were strongly species-specific, even though the three species share a relatively similar ecology and lifestyle^29^, illustrating the difficulty of projecting how biodiversity will be affected by continued climate change. We detected stabilizing selection in the Apollo and a negative association of temperature utilization with lifespan in the Large Blue, both of which are highly thermophilic^65,66^, at this high-latitude and relatively cold study site is alarming. Climate change allows many species to spread northward, but where can these highly specialized butterflies escape continued warming?

To allow for firm conclusions regarding long-term responses and evolvability, an important next step is to determine whether the extensive interindividual variation and intraindividual flexibility in temperature utilization indicated by our results have a genetic basis and whether they are influenced by conditions experienced during the egg, larval or pupal stages. Future investigations should also investigate how changing, and extreme temperatures affect reproductive success, mortality of early life stages, recruitment, and population dynamics^68,69^.

Finally, studies that examine whether the patterns, underlying drivers, and fitness consequences of variation in temperature utilization documented in this study are different from those in other populations of the same species that occupy areas with different environmental and ecological conditions, particularly with regards to the seasonality of temperature regimes, may advance our understanding of the evolution of biodiversity and aid the development of strategies for its conservation.

## Supplementary Information

Supplementary Information.

## Data Availability

Data used in the preparation of this manuscript can be accessed in the database www.artportalen.se.
