# Supplementary material for: Differences in phenology, daily timing of activity, and associations of temperature utilization with survival in three threatened butterflies
Source: Sci Rep. 2022 May 9;12:7534. doi: 10.1038/s41598-022-10676-0 (PMC9085768; doi:10.1038/s41598-022-10676-0)
Supplement: Supplementary file 1 — Supplementary Information. [file 41598_2022_10676_MOESM1_ESM.docx]

**Supporting Information**

**Table S1:** Pearson’s correlation coefficients between the eleven temperature loggers located in the study area. For all correlations, the P-value is <0.001.


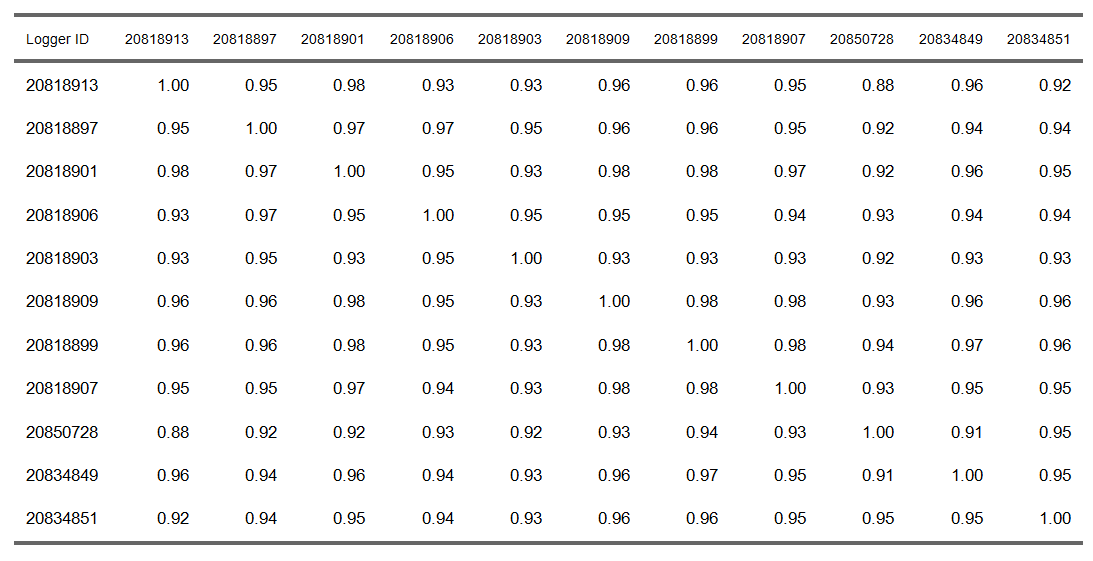


**Table S2:** Correlation between Number of times captured, temperature range, and lifespan for the three studied species.


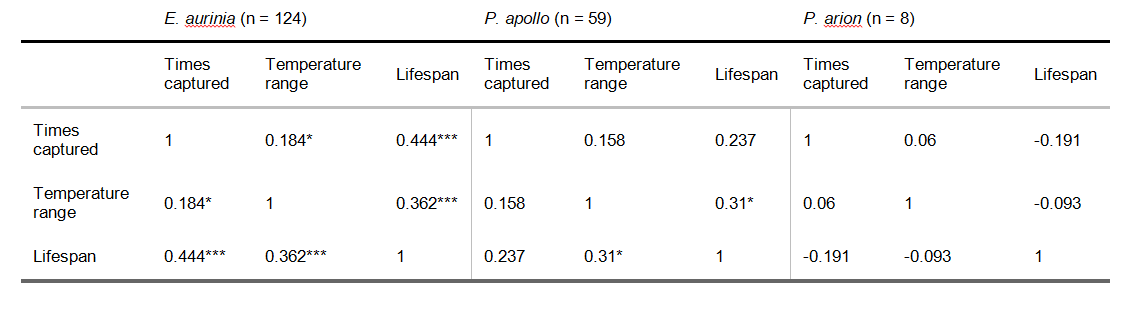


**
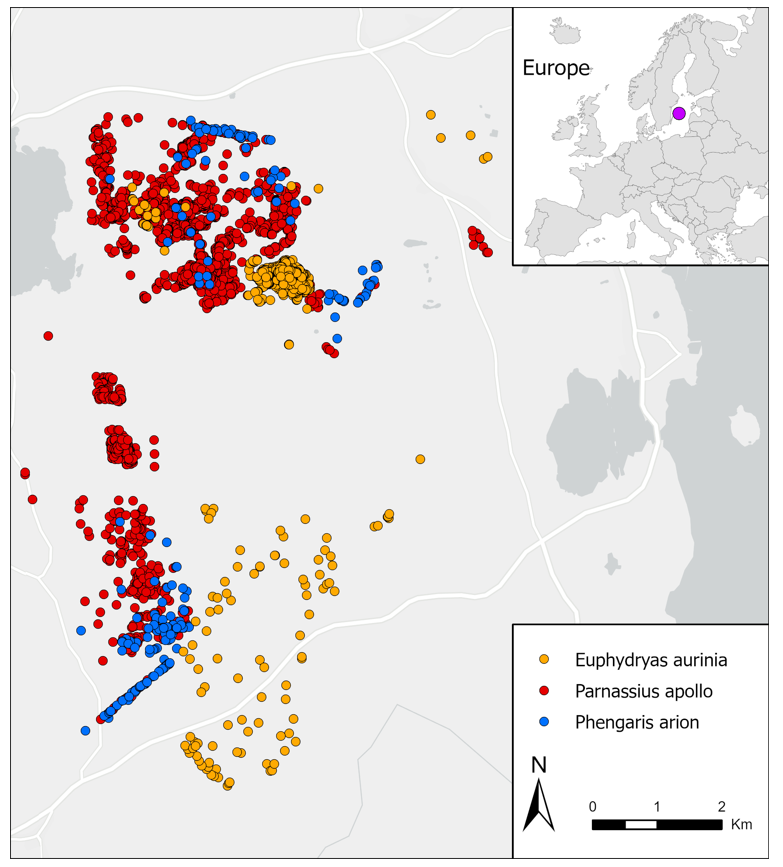
**

**Fig. S1.** Map of the study area on Gotland in southeastern Sweden with the marked individuals of the three species. Map created in ArcGIS pro version 2.8.


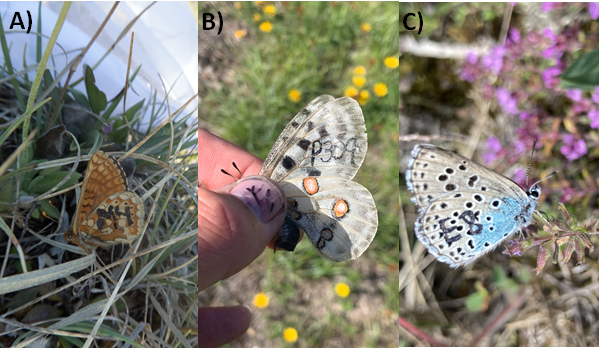


**Fig. S2.** Individually marked Marsh Fritillary (*Euphydryas aurinia*) (A), Apollo *(Parnassius apollo)* (B)*,* and Large Blue (*Phengaris arion)* (C). Photographs by Markus Franzén.


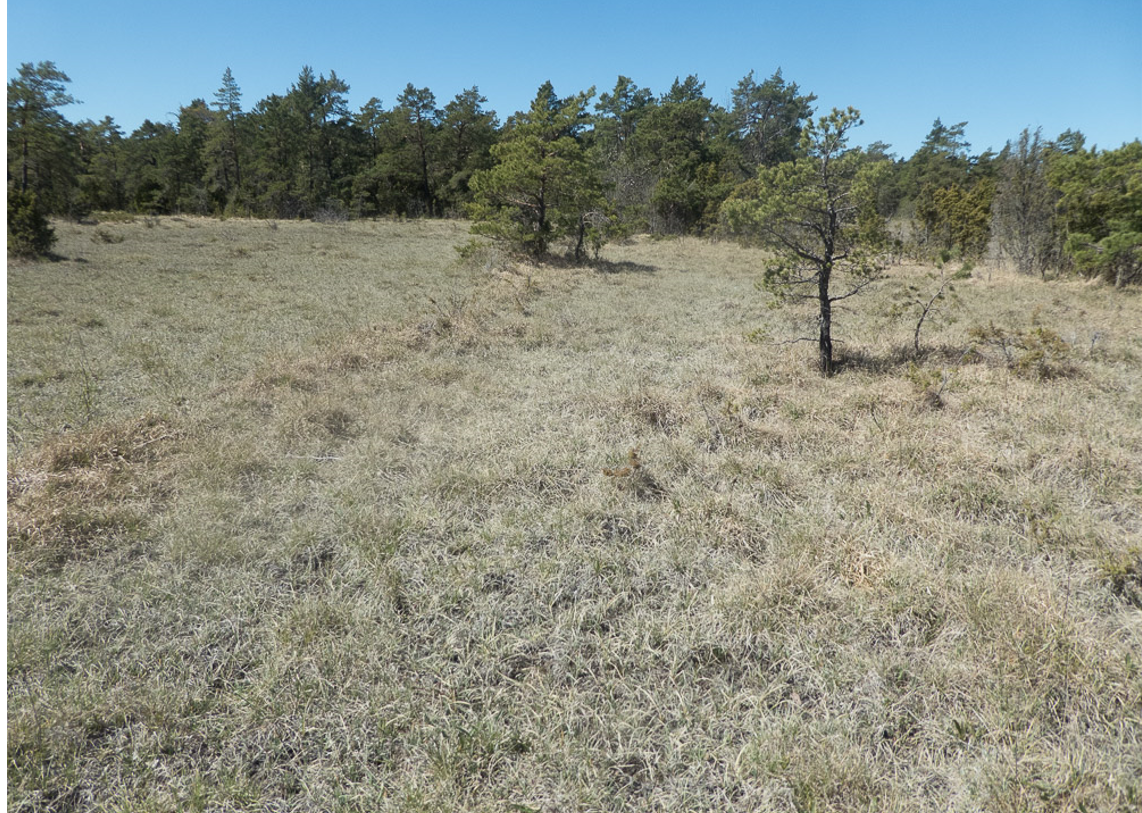

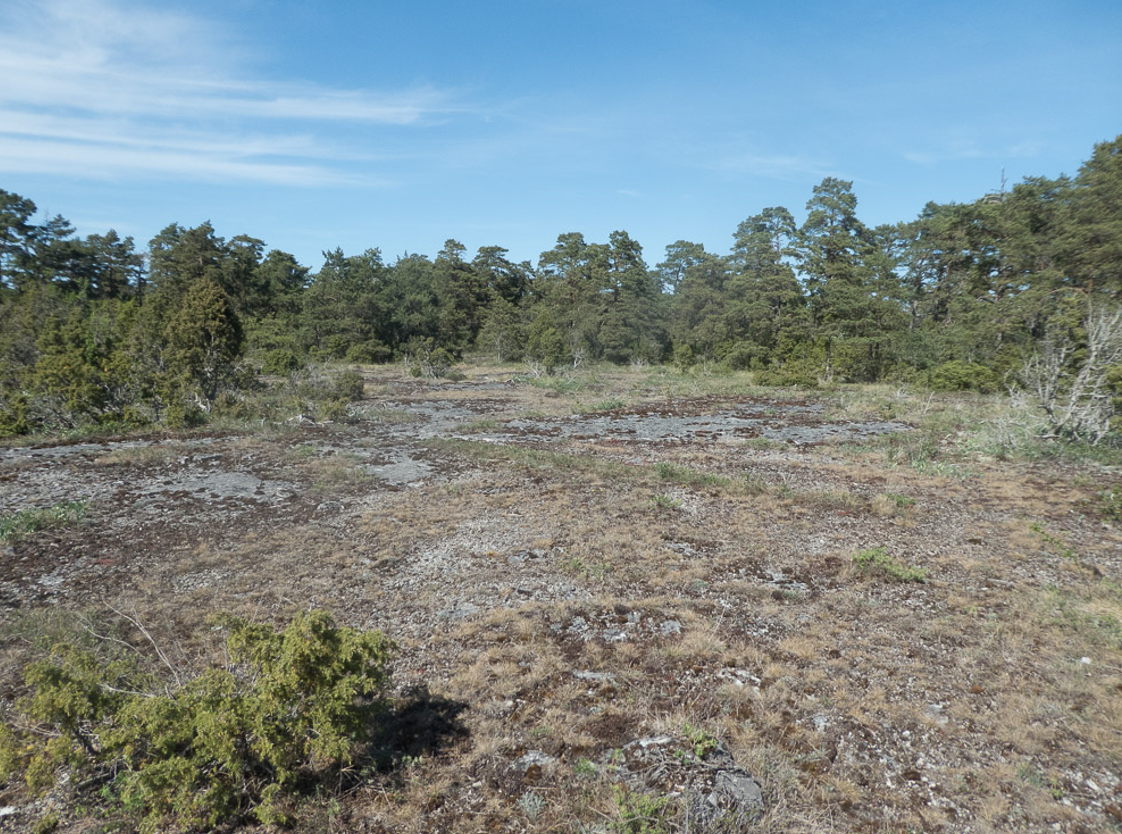
**
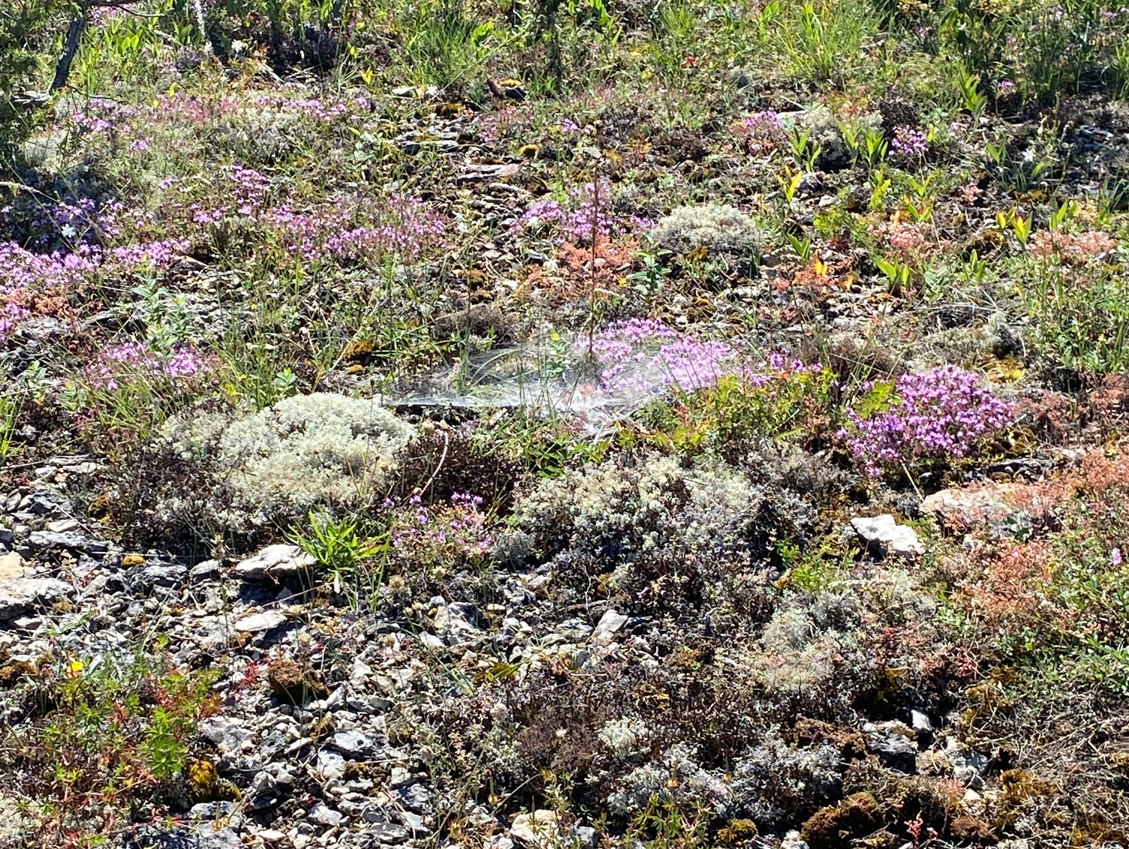
**

**Fig. S3.** Habitat on Gotland in southeastern Sweden for Marsh Fritillary (*Euphydryas aurinia*) (A), Apollo *(Parnassius apollo)* (B)*,* and Large Blue (*Phengaris arion)* (C). Photographs by Markus Franzén.


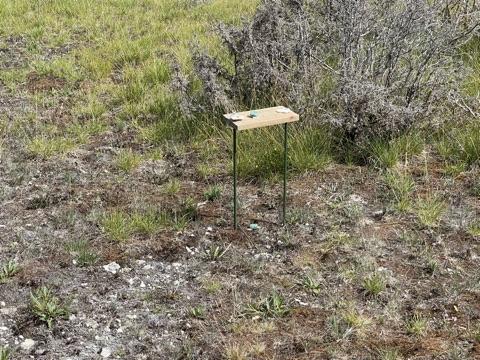


**Fig. S4.** The temperature logger HOBO MX2202 pendant wireless temperature/light data loggers were placed in the shade below the board 0.5 m above ground. Photograph by Markus Franzén.


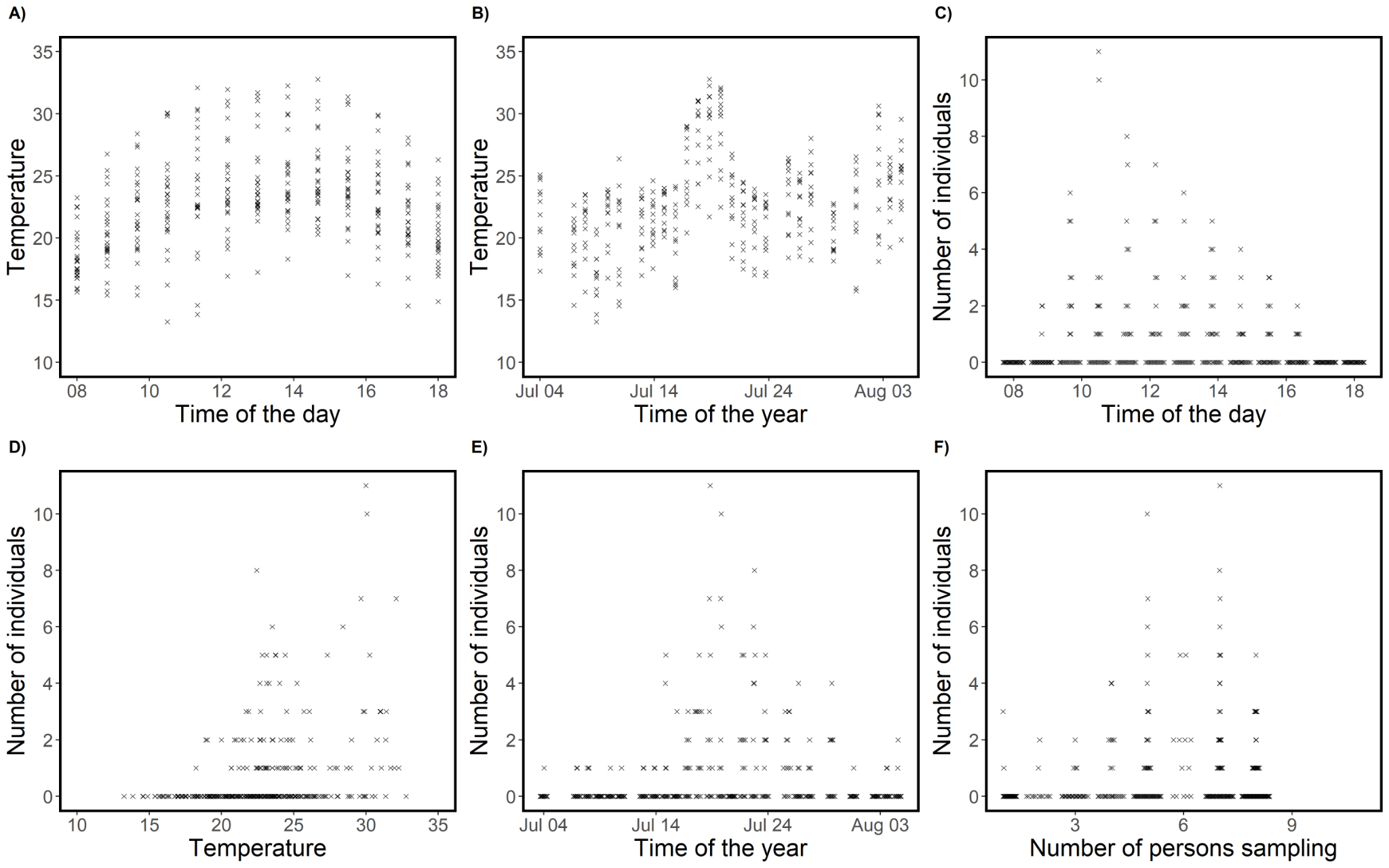


**Fig. S5.** Raw data for the Large Blue (*P. arion)*. A) The recorded hourly temperatures relative to the time of the day. B) the recorded hourly temperatures relative to the flying period. C) The number of individuals caught relative to the time of the day. D) the number of individuals caught relative to the hourly temperature. E) The number of individuals caught relative to the flying season. F) The number of individuals caught depends on the number of people sampling.


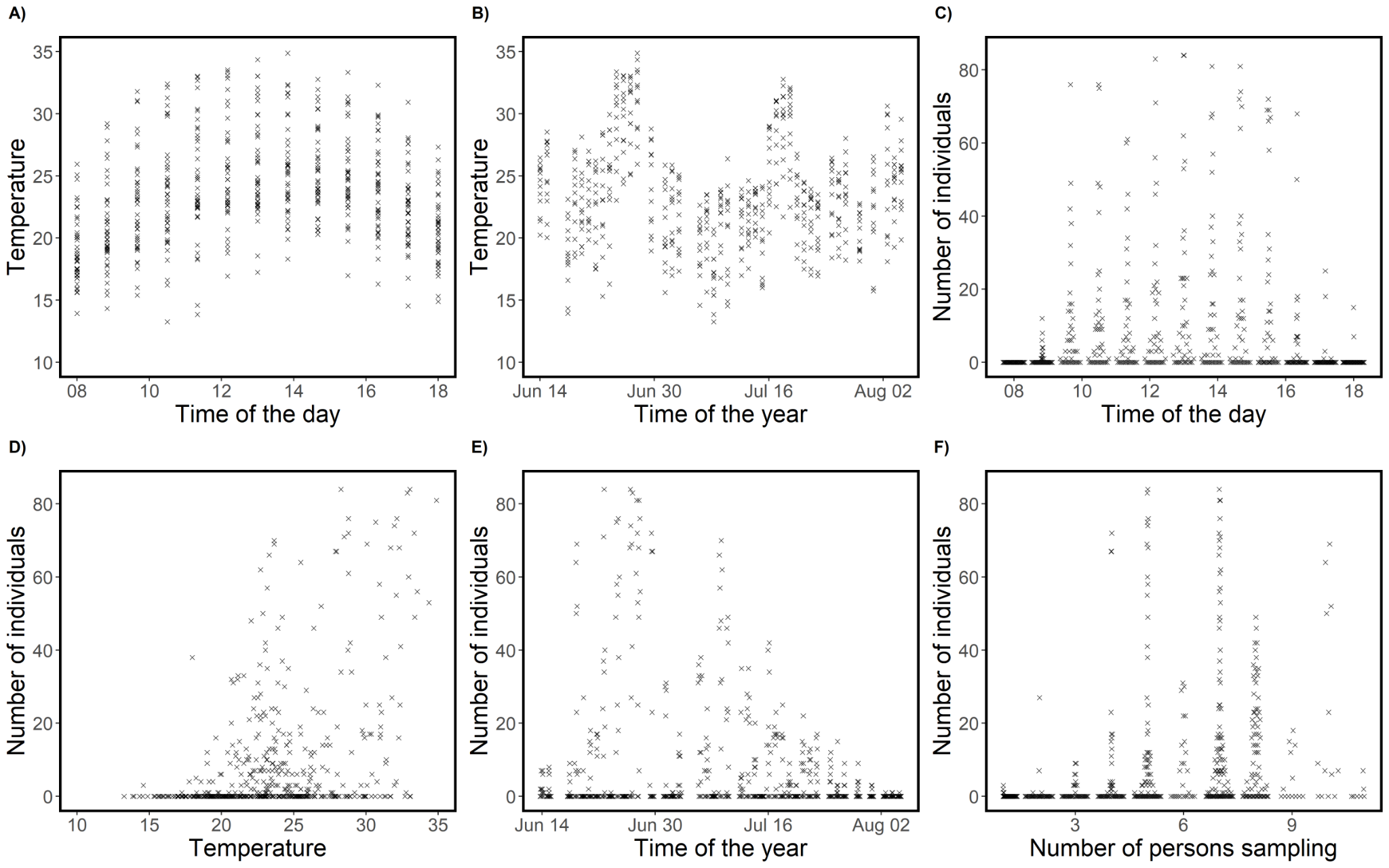


**Fig. S6.** Raw data for Apollo (*P. apollo*) The recorded hourly temperatures relative to the time of the day. B) the recorded hourly temperatures relative to the flying period. C) The number of individuals caught relative to the time of the day. D) the number of individuals caught relative to the hourly temperature. E) The number of individuals caught relative to the flying season. F) The number of individuals caught depends on the number of people sampling.


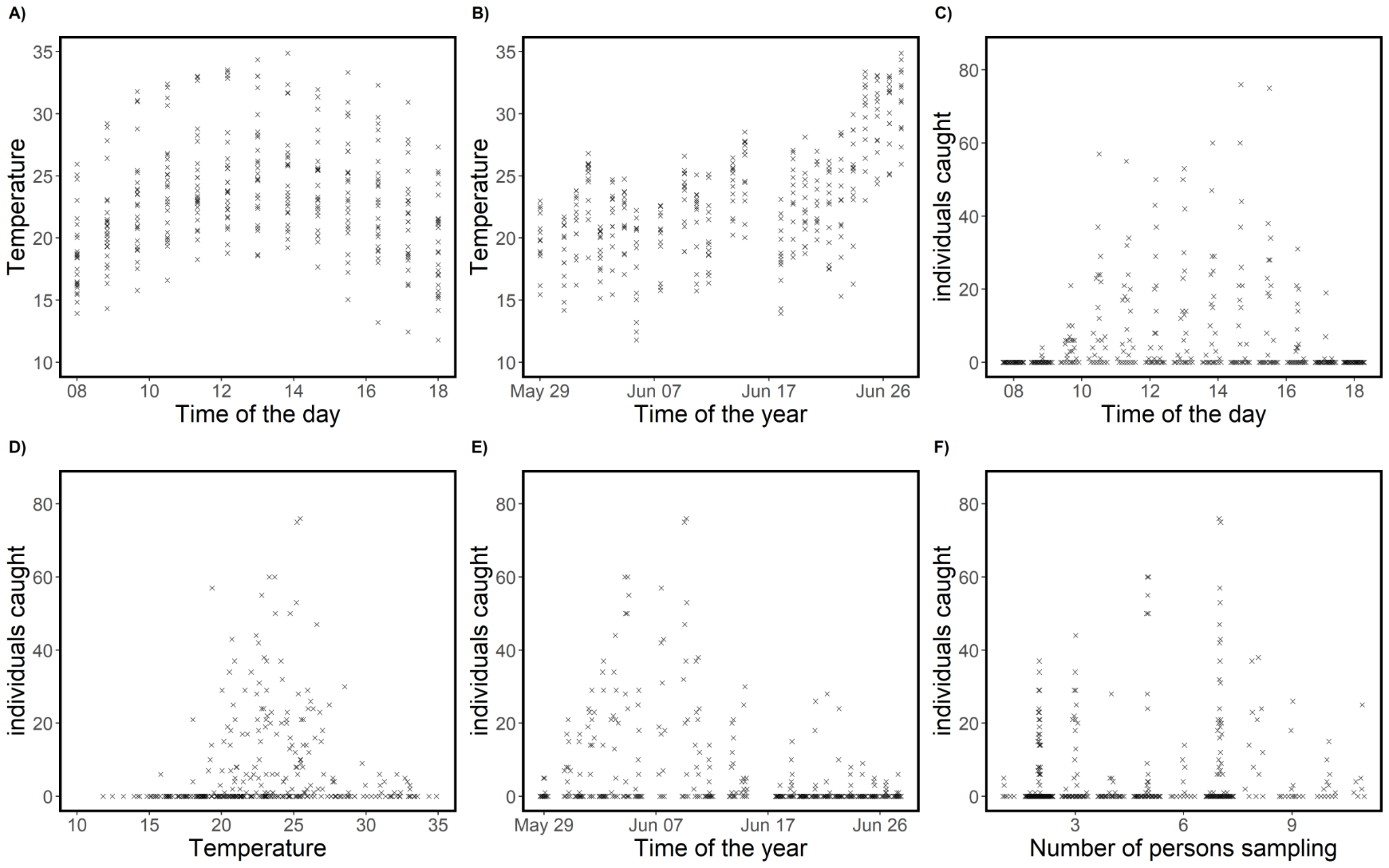


**Fig. S7.** Raw data for Marsh Fritillary (*E. aurinia)*. A) The recorded hourly temperatures relative to the time of the day. B) the recorded hourly temperatures relative to the flying period. C) The number of individuals caught relative to the time of the day. D) the number of individuals caught relative to the hourly temperature. E) The number of individuals caught relative to the flying season. F) The number of individuals caught depending on the number of people sampling.


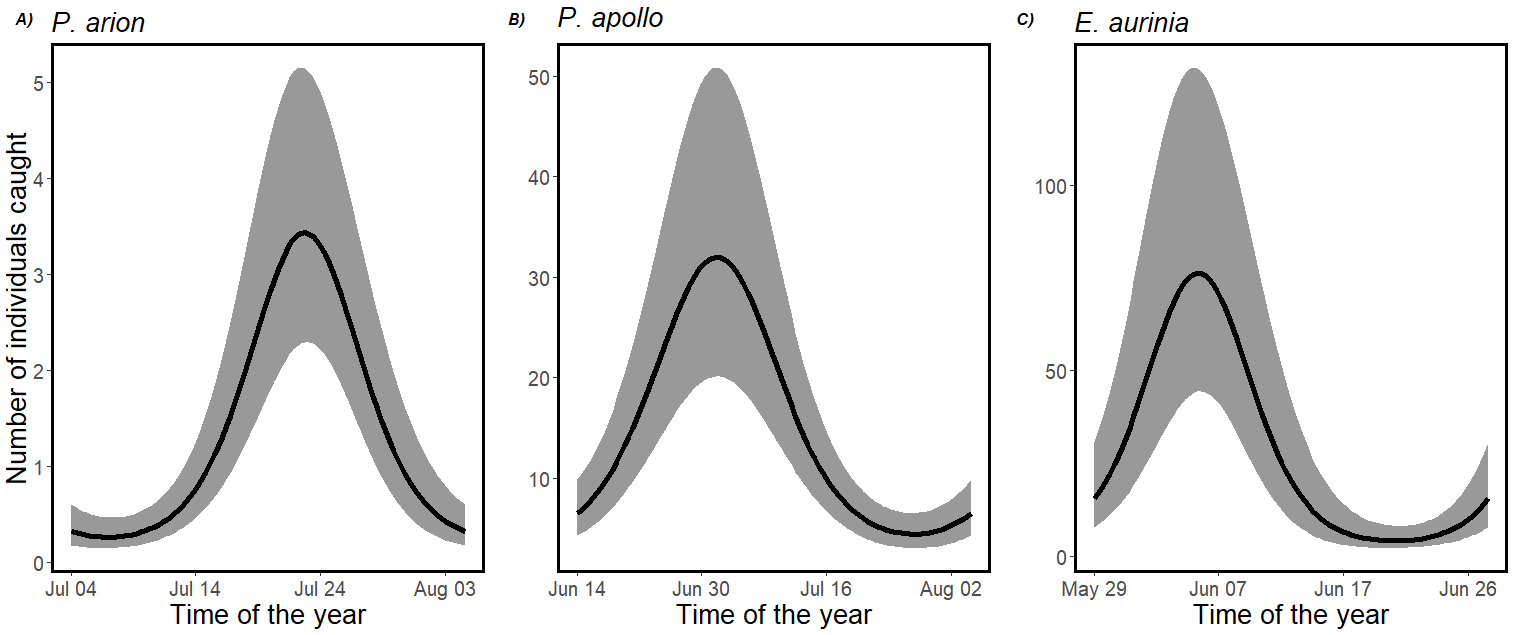


**Fig. S8**. Predicted effects of linear regression models for the activity over the flying period for *E. aurinia*, *P. arion,* and *P. apollo*.


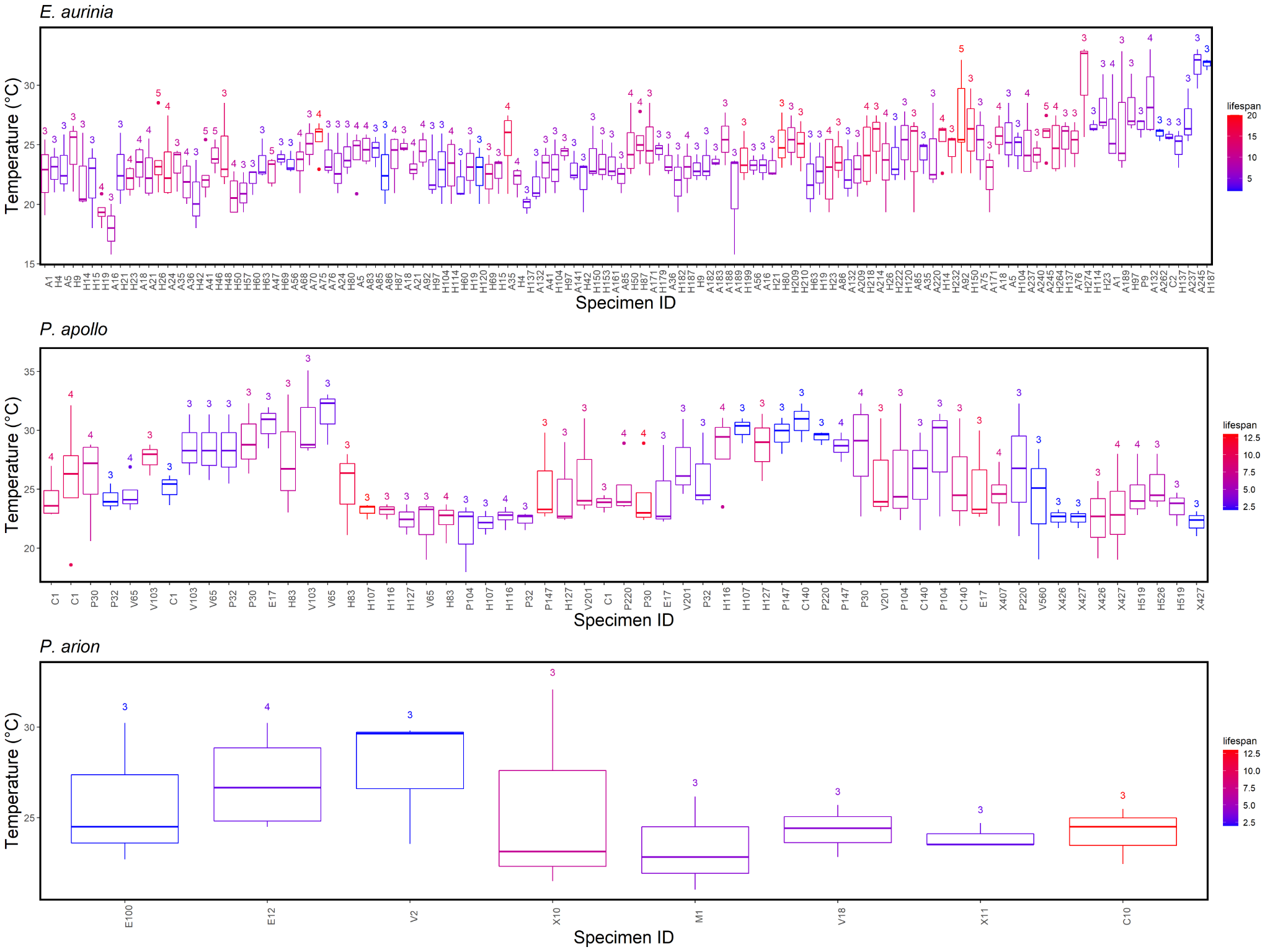


**Fig. S9.** Variation in intra-individual temperature preferences among individuals representing three butterfly species. Data on ambient temperature during activity for marked individuals that were caught on at least three different days. The number of special days the individual was caught is displayed at the mean activity temperature. Individuals are sorted along the horizontal axis according to the date of the first capture. Colors indicate differences in lifespan, from short (blue) to long (red). The boxplots show median, quartiles, and extreme values.


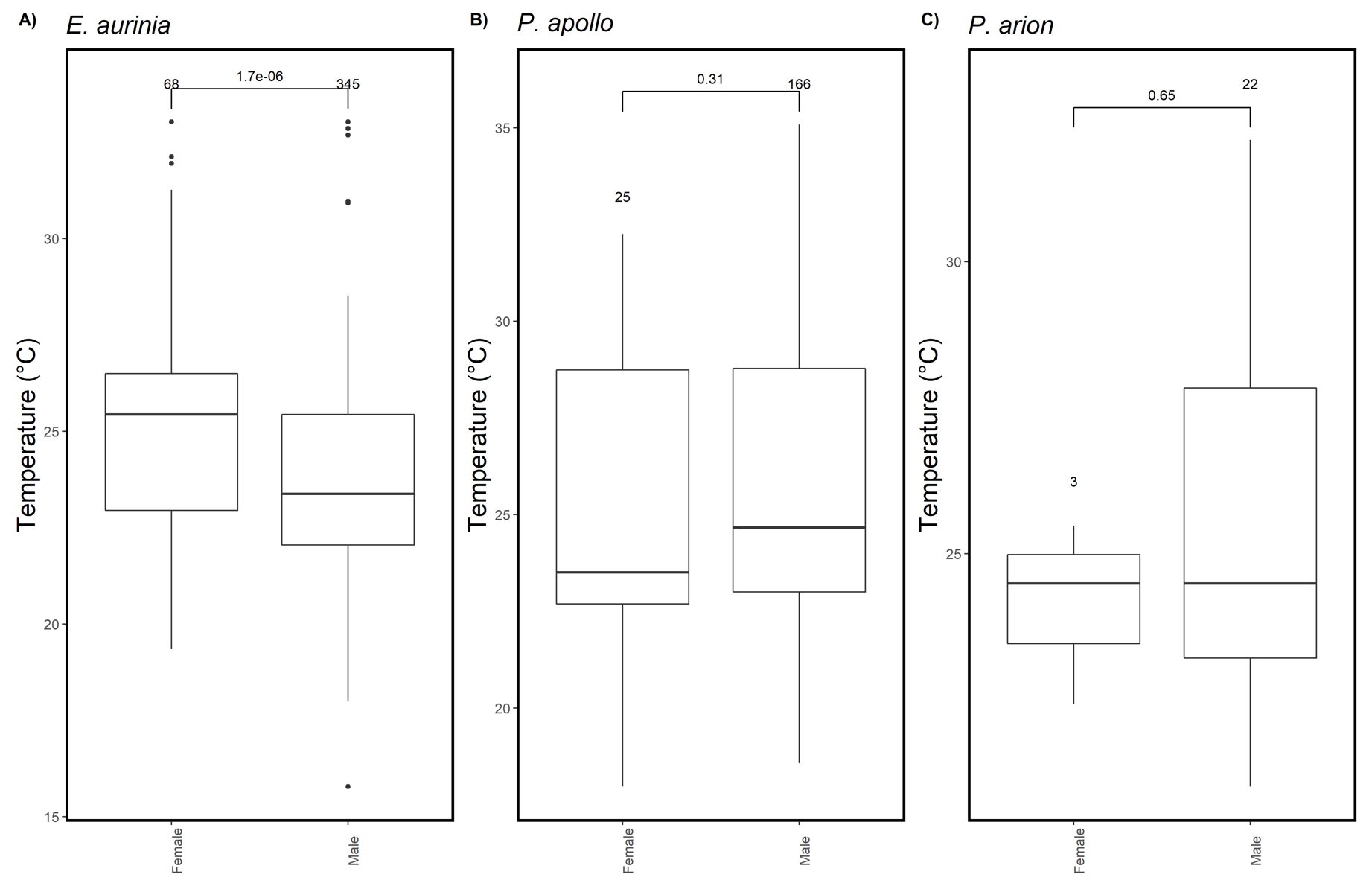


**Fig. S10.** Variation in intra-individual temperature preferences among individuals between sexes in the three butterfly species. The boxplots show median, quartiles, and extreme values.
